# Commentary: Learning Students' Given Names Benefits EMI Classes

**DOI:** 10.3389/fpsyg.2020.01625

**Published:** 2020-08-11

**Authors:** Kevin K. Jepson

**Affiliations:** College of the North Atlantic-Qatar, Doha, Qatar

**Keywords:** education, engagement, positive, pronunciation, names, success

Murdoch et al. (2018) contributed to an important yet inconspicuous concept associated with teaching—using students' given names. The authors' article focused on the use of English as the medium of instruction (EMI) in university classes in Korea and the use of student names as a precursor to student engagement and satisfaction and to success in the classroom.

Murdoch, Lim, and Kang juxtaposed the conflict students face learning subject material with that of infrequent teacher–student communication in EMI classes, which hampered the possibility of teachers learning the students' names. The authors' survey instrument examined the instructors' use of names, the students' perceptions of that use, and the influence of such use to motivate learning. The authors found that students who had previous EMI learning experiences felt more valued when instructors used their names. The principal concern, among reasons for negative perceptions of student name use, was incorrect pronunciation. However, when learning subject matter in English, I believe that a passive learning of Western cultural norms also occurs. The realities of an autonomously leaning classroom dynamic should therefore promote individual autonomy.

Deci and Ryan (2000, p. 229) postulated that self-determination theory (SDT) rests on self-socialization, and while it is counter-intuitive for instructors to guide student self-determination (ten Cate et al., 2011, p. 961), initial engagement can be created and continued by simply learning and then using student names. Boulton et al. (2019, p. 2) posited that defining and then connecting engagement to success requires a “holistic view of student motivations and appropriate measures of outcomes.” As such, instructors should take the “indirect or enabling” perspective of positive psychology to determine the favorable conditions that enable students to be more optimistic and content with their learning environment and then help to make those conditions a reality (Csikszentmihalyi, 2009, p. 204). The inclusive purpose of positive psychology as postulated by Seligman et al. (2005, p. 413) is being that of positive emotion, engagement, and meaning fitting well into a student success model of achieving educational goals. Commitment to learning is a vital factor in promoting student success, and student engagement is the key to generating and maintaining interest in education. As rapport is developed, so too is engagement. Without meaningful engagement, students may exhibit more individual attention-seeking behaviors, resulting in a more negative response from teachers (Swinson and Harrop, 2012, p. 68).

Glenz (2014, p. 21) posited that audibly using names leads to an atmosphere of confidence and encouragement. Indeed specific brain activation when hearing one's name is equal to that of an introspective analysis of personal characteristics; self-representation occurs in response to hearing one's own name (Carmody and Lewis, 2006, p. 4). Smith and Malec (1995, p. 281) reasoned that student contribution was noticeable when educators learned and used their names. Pearson and Lucas (2011, p. e672) posited that being visually recognized by name enabled deeper learning and engagement. Students felt more valued in the course and more than just a face in the crowd when instructors knew their names (Cooper et al., 2017, p. 10). Indeed “learning and self-esteem are heightened when individuals are in respectful and caring relationships with others who see their potential, genuinely appreciate their unique talents, and accept them as individuals” (Sanghani et al., 2013, p. 251).

Whereas, intrinsic motivation occurs in the absence of external rewards, it “depends on ambient supports for basic psychological needs” (Di Domenico and Ryan, 2017, p. 2). Teaching in Qatar has shown me that students have a deep appreciation for the feeling of community in the classroom and for teachers who learn their names despite any difficulties in pronunciation. Accordingly, Bitner (2017, p. 18) advised trying to learn names before beginning course instruction to convey a genuine interest in one's students. In the Muslim world, having a name that carries significance is a mark of respect and pride (Duckett, 2000, p. 29). Additionally, Mirza (2017, p. 43) noted that writing Muslim names correctly in English is crucial. In a study of Arabic-speaking immigrants in Canada, Pennesi (2016, p. 56) found that proper pronunciation of Arabic names equals that of respectful treatment.

Sanghani et al. (2013, p. 251) described the use of positive psychology in schools as facilitating students' growth and achieving their highest self. I also believe it is the educator's goal to see classrooms of engaged and engaging students rather than “students who are languishing” (White, 2016, p. 3). Csikszentmihalyi (2009, p. 209) warned of children being raised in negative environments, sitting in classes and completing tasks without having an objective in mind. It is worth noting that “wellbeing is of equal priority to academic learning in developing the whole student” (Waters, 2011, p. 84). In this way, as positive psychology is about enabling satisfaction in lives, positive education “first benefits teachers personally, and then benefits them again by making it easier to get students to engage with and persist in the work they need to master academic material” (Sanghani et al., 2013, p. 252).

To conclude, Murdoch et al. (2018) have presented an important point—the positive impact of using students' names within an EMI environment. While the authors correctly link success, motivation, and attitude into a three-pronged, symbiotic relationship, they noted that some students objected to the use of their names if mispronounced. However, using students' names aligns with the positive education principle which is “to create a school culture that supports the caring, trusting relationships that empowers the whole school system” (Sanghani et al., 2013, p. 252). Commitment to a student-centered, active-learning classroom provides an environment for engaged students to be more optimistic and confident in their prospects (Hunter and Csikszentmihalyi, 2003, p. 27).

Waters (2011, p. 77) postulated that positive education promotes positive emotions and constructive bonds. Indeed high-quality engagement supports student learning outcomes (Wang et al., 2019, p. 1088). Fear of mispronouncing names should not be an excuse to disregard the learning and using of student names. Should instructors not call students by name, we have failed in our efforts to include and engage. As a teacher with domestic and overseas teaching experience, I believe that using students' names makes students feel included, strengthens the connection between teachers and students, and creates a positive learning environment which reinforces learning.

## Author Contributions

KJ initiated and drafted the general commentary. The sole author approved the final version of the manuscript for submission.

## Conflict of Interest

The author declares that the research was conducted in the absence of any commercial or financial relationships that could be construed as a potential conflict of interest.
